# Assessment of Organophosphate Pesticides Exposure in Men with Idiopathic Abnormal Semen Analysis: A Cross-Sectional Pilot Study

**DOI:** 10.22074/IJFS.2020.134650

**Published:** 2021-06-22

**Authors:** Induja Manikandan, Sushmita Bora, Prashant Shankarrao Adole, Chitra Thyagaraju, Rajesh Nachiappa Ganesh

**Affiliations:** 1 Department of Biochemistry, Jawaharlal Institute of Postgraduate Medical Education and Research, Pondicherry, India; 2 Department of Obstetrics and Gynecology, Jawaharlal Institute of Postgraduate Medical Education and Research, Pondicherry, India; 3 Department of Pathology, Jawaharlal Institute of Postgraduate Medical Education and Research, Pondicherry, India

**Keywords:** Acetylcholines terase, Male Infertility, Pseudocholines terase, Organophosphate Pesticides

## Abstract

**Background:**

Because of the widespread use of organophosphate (OP) pesticides in agriculture, they are major environmental contaminants in developing countries. OP pesticides decrease sperm concentration and affect its quality, viability, and motility. Studies have demonstrated the association between abnormal semen analysis and OP pesticides exposure among the high-risk population. As there is limited data on the percentage of OP pesticides exposure, the study aimed to determine the OP pesticides exposure in Southern Indian men with idiopathic abnormal semen analysis and find the possible source of their OP pesticides exposure.

**Materials and Methods:**

In this cross-sectional pilot study, fifty men with idiopathic abnormal semen analysis as cases and fifty men with normal semen analysis as controls were recruited. Detailed history was taken and general and systemic examinations were carried out. OP pesticides exposure was determined by assessment of pseudocholinesterase and acetylcholinesterase levels and urinary OP pesticides metabolites dialkyl phosphate (DAP) consisting of dimethyl phosphate (DMP), diethyl thiophosphate (DETP), and diethyl dithiophosphate (DEDTP).

**Results:**

Cases had statistically significantly lower levels of pseudocholinesterase (5792.07 ± 1969.89 vs. 10267.01 ± 3258.58 IU/L) (P=0.006) and acetylcholinesterase [102.90 (45.88-262.74) vs. 570.31 (200.24-975.30) IU/L] (P=0.001) as compared to controls. Cases had a statistically significantly higher percentage of urinary DAP positivity as compared to controls (80 vs. 38%, P<0.0001). Hence, cases had a significantly higher percentage of OP pesticides exposure as compared to controls (20 vs. 4%, P=0.015). OP-exposed cases had significantly higher urinary DETP and DEDTP levels as compared to OP non-exposed cases. Also, urinary DETP and DEDTP levels were significantly negatively associated with sperm concentration, motility, and normal morphology among OP-exposed cases.

**Conclusion:**

Southern Indian men with idiopathic abnormal semen analysis had a significantly higher percentage of OP pesticides exposure as compared to men with a normal semen analysis.

## Introduction

Infertility as one of the major public health problems is defined as “a failure in achieving a clinical pregnancy after 12 or more months of regular unprotected sexual intercourse” as per the World Health Organization (WHO) (1). According to the WHO, 45-52.6 million married couples were suffering from infertility worldwide in 2010 (2). The prevalence of infertility among Indians was ranging from 3.9 to 16.8% as estimated by the WHO (3). As per the report of a multicentric study by the WHO, 20% of cases of infertility were due to male factors, 38% due to female factors, 27% due to both partners, and 15% cases of infertility were idiopathic. In India, nearly 50% of cases of infertility were due to the reproduction anomaly or disorders in males and in 25% of cases, no detectable causes were found and it was considered idiopathic (4). Male infertility is rising in society and its causes are multifactorial. Many studies have shown a declining trend in the semen quality and sperm count among the population (5, 6). A study conducted in the Indian population over the past 37 years has shown a decline in sperm count and motility, and altered sperm morphology with time (7). No clear cause has been found for the decline of semen quality, but it might be due to environmental, dietary, or other unknown causes (5).

Organophosphate (OP) pesticides are synthetic chemicals used worldwide for controlling domestic and agricultural pests. Pesticides used to control pests and weed on crops are registered under the central insecticides board and registration committee (CIBRC), which comes under the Ministry of Agriculture and Farmer welfare. Section-3 of the Insecticides Act, 1968 has registered around 30 OP pesticides, which are in use in India (8). OP pesticides like monocrotophos, phorate, quinalphos, malathion, chlorpyrifos, diazinon, methyl parathion, ethion, and so on, used extensively in India, were already banned or severely restricted in the USA and Europe. The OP pesticides are associated with severe toxicity, contributing to more than 80% of pesticides-related hospitalization in India (9). OP pesticides cause phosphorylation of acetylcholinesterase resulting in acetylcholine accumulation in synapses. OP pesticides affect reproduction function by reducing acetylcholinesterase activity in the brain, and influencing gonads. OP pesticides like parathion and methyl parathion have a structure similar to hormones like estrogen, thus altering genes expression by interacting with hormone receptors. OP pesticides alter the hypothalamic-pituitary (HPO), pituitary-thyroid, and pituitary-adrenal axes and serum prolactin levels. OP pesticides affect spermatogenesis by damaging the Sertoli and Leydig cells and increasing their apoptosis (10). A toxicological study demonstrated that OP pesticides cause low sperm concentration by affecting germ cell proliferation and damaging the seminiferous epithelium (11). Also, OP pesticides disturb sperm motility by disturbing its tail assembly proteins or ATP synthesis (12). Concerning the association between semen parameters and OP pesticides exposure among agricultural workers, pesticide sprayers, and workers in pesticides manufacturing industries, several studies concluded that there was a decrease in sperm concentration, motility, viability, and normal morphology due to OP pesticides exposure (13-18). There has been contamination of agricultural soil, sediment, and water by various OP pesticides throughout India (9). Hence, subtle OP pesticides exposure is occurring among human beings through food, water, air, tainted breast milk, playing in the field, or skin contact. 

Most of the studies were done on high-risk populations to find out potential associations between OP pesticides exposure and alteration in semen parameters. However, there is limited data available in the literature to say that environmental OP pesticides exposure associates with abnormal semen parameters among the general population. Therefore, the present study aimed to assess the environmental OP pesticides exposure among Southern Indian men from Pondicherry and surrounding districts of Tamil Nadu, like Tindivanam, Villianur, Chennai, and Villupuram with idiopathic abnormal semen analysis by measuring pseudocholinesterase and acetylcholinesterase levels and urinary OP pesticides metabolites. The objectives of the study were to compare environmental OP pesticides exposure between men with and without idiopathic abnormal semen analysis and to determine possible sources of OP pesticides exposure by comparing percentages of farmers, rural population, smokers, undergraduates, lower socioeconomic status, vegetarians, people using underground water source, and alcoholics

## Materials and Methods

### Study design and population

This cross-sectional pilot study was conducted in the Jawaharlal Institute of Postgraduate Medical Education and Research (JIPMER) hospital, Puducherry, 605 006 from January 2018 to July 2019 after obtaining the approvals from Institute Research Council and Institute Human Ethics Committee (JIP/IEC/2017/0351 dated November 27, 2017). All Southern Indian men, 25 to 45 years old, from Pondicherry and surrounding districts of Tamil Nadu, like Tindivanam, Villianur, Chennai, and Villupuram, who was attending the JIPMER Infertility Clinic, Pondicherry for the inability of their spouse to conceive after 1 year of unprotected sexual intercourse, were recruited. Written informed consent (both in English and Tamil) after explaining the purpose and the procedure of the study, was obtained from all the participants. After obtaining the consent, detailed history of the patient was taken including his occupation, location, source of water, history of any medication, or surgery, and other demographic factors. General and systemic examinations were carried out and the scheduled date for semen analysis was given. On the scheduled day, a semen sample, 10 ml of urine, and 5 ml of blood were collected under sterile conditions. Fifty men who had abnormal semen analysis (sperm count ≤15 million/ml, sperm motility ≤40% and sperm morphology ≤4% normal) as per WHO criteria 2010 (4) with no identifiable pathology, were recruited as cases. Cases with underlying pathology such as varicocele, history of diabetes, cardiovascular or thyroid disorders, tuberculosis, testicular carcinoma, obstruction or congenital bilateral absence of vas deferens, or use of lipid-lowering drugs, were excluded. Fifty men with normal semen analysis were recruited as controls. So, it was a pilot study with 50 cases and 50 controls, which was accepted by the institute research council. 

### Criteria for analysis of anthropometry, alcohol use, smoking, and socioeconomic status

The height, weight, body mass index (BMI), and waist circumference were measured by the same observer. The nearest half-kilogram for body weight and half-centimeter for height were recorded. The waist circumference was determined by measuring the shortest point below the lowermost rib cage margin and the iliac crest and was recorded to the nearest half-centimeter. BMI was calculated as weight (kg) divided by the square of height (m). Being alcoholic was defined by the consumption of at least two drinks per day. A standard drink was equal to either 10g/12.7 ml of pure alcohol, 330 ml of beer, 100 ml of wine, or 30 ml of straight spirits or liquor like gin, rum, vodka, or whiskey. Being smoker was defined as having a history of smoking over the past one year irrespective of the number of cigarettes per day (19). Being vegetarian was defined as eating animal products either never or rarely (less than once per month), consuming dairy products and eggs, but eating meat/ fish less than once per month or who ate fish more than once per month, but other meats less than once per month (20). The socioeconomic status was assessed based on the Kuppuswamy criteria (21).

### Semen collection and analysis

Semen was collected in a sterile container by masturbation in a private room near the laboratory. Participants were asked to abstain from ejaculation for 3 days before the scheduled date of appointment. Semen volume was measured in a graduated cylinder. Sperm counts were determined on two separate drops of semen using a Neubauer haemocytometer. If the sperm counts determined in the two drops of semen differed by 10% or more, then the count was determined in the third drop of semen. In this case, the sperm count from the first two samples which was closest and within 10% of the third sample count was retained. Sperm count was calculated as the average of the two sperm counts. Sperm motility was determined microscopically in two 10-μl drops from the semen sample. Slides were prepared and observed for altered sperm morphology (22). 

### Estimation of pseudocholinesterase and acetylcholinesterase levels

Serum pseudocholinesterase levels were measured by sandwich enzyme-linked immunosorbent assay kit from LifeSpan Biosciences, Inc. according to the manufacturer’s instructions. The intra-assay and inter-assay coefficient of variation (CV) for serum pseudocholinesterase was less than 10 and 12%, respectively. Serum acetylcholinesterase levels were measured by the Ellman method in which thiocholine, produced by acetylcholinesterase, reacted with 5,5-dithiobis (2-nitrobenzoic acid) to form a colorimetric (412 nm) product, proportional to the acetylcholinesterase activity present. One unit of acetylcholinesterase is the amount of enzyme that catalyzes the production of 1.0 mmole of thiocholine per minute at pH=7.5 at room temperature (23).

### Sample preparation and gas chromatography-mass spectrometry

Ten milliliters of urine were collected and stored at -80°C until further analysis. All urine samples were thawed and mixed by a vortex. Cleaning of urine sample and derivatization of alkyl phosphate were done as per Hemakanthi De Alwis et al. (24). A Trace GC Ultra equipped with AI 3000 Auto-Injector (Rodano, Italy) and ITQ 900 mass spectrometer from Thermo Scientific (Austin, USA) was used for analysis with a constant flow rate of 1.2 ml/minutes and Helium as a carrier gas. One microliter of a sample from the low volume insert was injected in splitless mode onto a Thermo Electron Corporation (Rodana, Italy) TR-5MS ([5%- phenyl]-polysilphenylene-siloxane) TRACE GC capillary column (30 m, 0.25 mm, 0.25 μm) using the autosampler. The GCMS protocol followed was as per Hemakanthi De Alwis et al. (24). All the standards for dimethyl phosphate (DMP), diethyl thiophosphate (DETP), diethyl dithiophosphate (DEDTP), Sulfotep, an internal standard and derivatization agent 2,3,4,5,6- Pentafluorobenzyl bromide were purchased from SigmaAldrich with a purity of ≥90%. The mass spectra of the pentafluorobenzyl esters of DMP, DETP, DEDTP, and Sulfotep was determined. The analysis was done on selective ion monitoring (SIM) mode. The retention time (RT), linearity, the limit of detection (LOD), and limit of quantification (LOQ) for DMP, DETP, DEDTP, and Sulfotep were detected. The data obtained were transferred to X-Calibur files and manually evaluated. The peaks of the samples processed were recognized using the RTs and confirmed by comparison with the analyte/Sulfotep ratio for the two ions of the analyte. Results were reported utilizing creatinine adjustment

### Organophosphate pesticides exposure criteria

Both the presence of DAP in urine and inhibition of pseudocholinesterase were mandatory for the patients to be labeled as OP pesticides exposed. Patients with DMP, DETP, and/or DEDTP detected in urine above the LOQ labeled as DAP positive. Proudfoot formula was used as a basis for the determination of inhibition of pseudocholinesterase. We considered 4621-11500 IU/L as normal level (> 50%), 2311-4620 IU/L as mild inhibition (20- 50%), 460-2310 IU/L as moderate inhibition (10-20%) and less than 460 IU/L as severe inhibition (less than 10%) (14).

### Statistical analysis

The normality of data was assessed by the Kolmogorov-Smirnov test. The distribution of categorical data such as socio-demographic status, occupation, being vegetarian, being smoker, being alcoholic, and people using underground water, are expressed as percentages. The continuous data such as semen parameters, pseudocholinesterase and acetylcholinesterase levels, and OP metabolites in urine are expressed as mean with standard deviation or median (interquartile range). Creatinine was analyzed in urine and all OP metabolites values were adjusted for creatinine. All OP metabolites concentrations were log-transformed for statistical analysis. Descriptive statistics for OP metabolites among exposed and non-exposed included the percent above the LOD, mean and standard deviation, geometric mean and standard deviation, ranges, and calculation of the 25^th^, 75^th^, and 90th percentile. OP metabolites concentration below the LOD was assigned a value equal to the LOD/√2 (25). Binary logistic regression was done to estimate relative odd of urinary OP metabolites among exposed and non-exposed groups after adjustment to age, the number of married years, height, weight, waist circumference, percentage of undergraduates, lower socioeconomic status, vegetarians, primary infertility and alcoholics. Spearman’s or Pearson’s correlation was assessed between semen parameters and urinary OP metabolites in the OP-exposed group. Normally distributed variables were compared using the student’s t test. Non-parametric parameters were compared by the Kruskal-Wallis H test. Statistical analyses were done using SPSS 10 software at a significance level of 5% and P<0.05 was considered significant.

## Results

General characteristics were compared between cases and controls (Table 1). Cases had a significantly higher percentage of farmers as compared to controls (44 vs. 18%, P=0.009). Similarly, cases had a significantly higher percentage of rural population as compared to controls (60 vs. 38%, P=0.045). Contradictorily, a high percentage of smokers was found among controls as compared to cases (28 vs. 10%, P=0.022). However, no significant difference was found between cases and controls in age, the number of married years, height, weight, waist circumference, percentage of undergraduates, lower socioeconomic status, being vegetarian, using underground water, primary infertility, and being alcoholic. Pseudocholinesterase levels (5792.07 ± 1969.89 vs. 10267.01 ± 3258.58 IU/L, P=0.006) and acetylcholinesterase levels [102.90 (45.88-262.74) vs. 570.31 (200.24-975.30) IU/L, P=0.001] were statistically significantly lower among cases as compared to controls. 

Forty out of 50 cases had urinary DAP positivity. Pseudocholinesterase (4917.65 ± 900.54 vs. 5496.97 ± 1515.90 IU/L, P=0.007) and acetylcholinesterase [88.00 (45.62-249.47) vs. 197.64 (54.57-262.74) IU/L, P=0.001] levels were significantly lower among 40 cases with urinary DAP positivity as compared to 10 cases with urinary DAP negativity. Out of the 40 cases with urinary DAP positivity, 10 (25%) cases had mild inhibition (4417 ± 200 IU/L) and 30 (75%) cases had normal pseudocholinesterase levels. Nineteen out of 50 controls had urinary DAP positivity. Out of the 19 controls with urinary DAP positivity, 2 (10.6%) men had mild inhibition and 17 (89.4%) men had normal pseudocholinesterase levels. However, all controls with DAP negativity had normal pseudocholinesterase levels.

Ten out of 50 cases had both inhibitions of pseudocholinesterase and urinary DAP positivity, hence they were labeled as OP pesticides-exposed. Two out of 50 controls had both inhibitions of pseudocholinesterase and urinary DAP positivity, hence they were labeled as OP pesticides-exposed. Cases had a significantly higher percentage of OP pesticides exposure in comparison with controls (20 vs. 4%, P=0.015). Also, cases with OP pesticides exposure had significantly higher urinary DETP and DEDTP levels as compared to cases without OP pesticides exposure (Table 2). Binary logistics regression showed that OP-exposed cases had significantly higher urinary DETP (OR=1.12, 95% CI=1.01- 1.26), DEDTP (OR=1.27, 95% CI=1.02-1.45) and DAP (OR=1.33, 95% CI=1.13-1.66) levels as compared to non-exposed cases after adjustment to age, the number of married years, height, weight, waist circumference, percentage of undergraduates, lower socioeconomic status, vegetarians, people using underground water, primary infertility and alcoholics (Table 3). Correlation analysis among OP-exposed cases showed that urinary DAP levels were significantly negatively associated with sperm concentration (P=0.001, r=-0.634), motility (P=0.001, r=-0.523), and normal morphology (P=0.001, r=-0.721).

**Table 1:** Comparison of general characteristics between cases and controls


Parameters	Men withidiopathic abnormal semenanalysis, cases (n=50)	Men with normal semen analysis,controls (n=50)	P value^*^

Age (Y)	34.94 ± 5.23	34.20 ± 6.61	0.479
Duration of marriage (Y)	5.49 ± 3.74	5.04 ± 2.53	0.889
Height (cm)	169.64 ± 12.21	168.76 ± 4.74	0.636
BMI (Kg/m^2^)	24.98 ± 4.44	24.65 ± 3.79	0.692
Wais‌t circumference (cm)	92.28 ± 16.03	86.90 ± 17.44	0.112
Undergraduates (%)	41 (82)	37 (74)	0.334
Farmers (%)	22 (44)	9 (18)	0.009
Lower socioeconomic s‌tatus (%)	17 (34)	8 (16)	0.068
Rural population (%)	30 (60)	19 (38)	0.045
Vegetarians (%)	7 (14)	7 (14)	1.000
Underground water source (%)	46 (92)	42 (84)	0.218
Primary infertility (%)	48 (96)	45 (90)	0.436
Smokers (%)	5 (10)	14 (28)	0.022
Alcoholics (%)	13 (26)	12 (24)	0.579
Semen volume (ml)	2.0 (1.5-2.5)	2.0 (1.5-2.5)	0.939
Sperm concentration (million/ml)	11.20 (3.00-46.25)	85.90 (65.45-121.90)	0.001
Sperm number (million/ejaculate)	26.95 (10.25-112.50)	127.75 (93.75-219.68)	0.001
Sperm motility (%)	8 (2-12.80)	53 (47-61)	0.001
Sperm morphology (%)	3 (2-4)	19.5 (17-23)	0.001
Pseudocholines‌terase (IU/L)	5792.07 ± 1969.89	10267.01 ± 3258.58	0.006
Acetylcholines‌terase (IU/L)	102.90(45.88-262.74)	570.31 (200.24-975.30)	0.001
DAP positivity (%)	40 (80)	19 (38)	<0.0001


Data are presented as mean ± SD, median (interquartile range) or percentage (%). BMI; Body mass index, DAP; Dialkyl phosphate, and *; Independent sample t test/ Kruskal-Wallis H test.

**Table 2:** OP pesticides metabolites levels between OP exposed and non-exposed cases


OP metabolites (selected ions)	LOD*/LOQ	Mean (SD)	GM (GSD)	Median	25^t^^h^ per	75^t^^h^ per	90^t^^h^ per	Range

DMP^*^ (306, 307)	10/33.4							
OP exposed		15.50 (11.43)	14.41 (89.66)	15.65	8.32	18.87	22.34	7.09-55.98
OP non-exposed		14.52 (6.87)	13.67 (76.67)	14.42	7.98	9.78	12.98	7.09-39.17
DETP^*^ (274, 350)	0.14/0.457							
OP exposed		46.78 (39.78)	32.45 (7.98)	35.26	11.24	55.67	88.65	0.14-146.67
OP non-exposed		26.56 (22.34)	11.46 (6.54)	15.50	8.45	38.87	67.54	0.14-80.67
DEDTP^*^ (366, 185)	3.06/10.20							
OP exposed		55.23 (46.32)	42.87 (9.45)	46.11	14.34	55.98	75.98	3.06-97.56
OP non-exposed		35.34 (30.21)	19.45 (8.67)	27.02	10.09	54.56	66.98	3.06-76.07


OP; Organophosphate, SD; Standard deviation, GM; Geometric mean, GSD; Geometric standard deviation, DMP; Dimethyl phosphate, DETP; Diethyl thiophosphate, DEDTP; Diethyl dithiophosphate, LOD; Limit of detection, LOQ; Limit of quantification, and * ; Samples below the LOD were defined as LOD/√2.

**Table 3:** Relative odd of OP metabolites after adjustment among OP exposed and non-exposed cases


OP metabolites	OR	CI	P value

DMP	0.65	0.23-1.43	0.363
DETP	1.12	1.01-1.26	0.004
DEDTP	1.27	1.02-1.45	0.004
DAP	1.33	1.13-1.66	0.001


OP; Organophosphate, DAP; Dialkyl phosphate, DMP; Dimethyl phosphate, DETP; Diethyl thiophosphate, DEDTP; Diethyl dithiophosphate, OR; Odd ratio, and CI; Confidence interval.

To find out the possible source of OP pesticides exposure, the general characteristics between OP-exposed cases and non-exposed cases were compared. Percentages of farmers and residing in a rural area were significantly higher in OP-exposed cases as compared to non-exposed cases. However, there was no significant difference in age, BMI, or waist circumference as well as percentages of men with undergraduate education, lower socioeconomic status, being vegetarian, using underground water, being smokers, and being alcoholics among OP-exposed cases as compared to non-exposed cases.

## Discussion

Our study reports that Southern Indian men with idiopathic abnormal semen analysis had a significantly higher percentage of OP pesticides exposure as compared to men with a normal semen analysis. Also, we found a significant correlation between urinary OP metabolites and semen parameters among OP-exposed cases.

As there is rampant use of OP pesticides in agriculture, their residues can be found in cooked meals, water, wine, fruit juices, refreshments, and so on. Also, washing and peeling cannot remove the OP residues completely (26, 27). Chronic, low-dose exposure to OP pesticides was found to be associated with neurodevelopmental problems in children, Parkinson's disease, metabolic syndrome, obesity, diabetes, reduced semen quality, reduced gestational age, reduced birth weight, and so on (28, 29). OP pesticides were found to affect the sperm quality directly or indirectly resulting in infertility and reproduction problems in the agricultural workers. OP pesticides act as endocrinedisrupting chemicals, alter the HPO axis, and impair spermatogenesis by damaging the Sertoli and Leydig cells (30). The general population is exposed to OP pesticides mainly through diet, inhalation of air, dermal absorption, and unintentional ingestion (31, 32).

Comparing general characteristics, we noticed that men with idiopathic abnormal semen analysis were mostly farmers and from the rural area as compared to men with a normal semen analysis. Our observations were consistent with those reported by Miranda‐Contreras et al. (14) who concluded that sperm count, motility, and membrane integrity among Venezuelan farmworkers were affected by occupational pesticides exposure. Also, Katole and Saoji (33) have reported a lower prevalence of primary infertility among urban populations. Dutta and Bahadur (34) showed that pseudocholinesterase and acetylcholinesterase levels were decreased among occupationally-exposed tea garden workers of the Northern part of West Bengal, India, similar to our observations. Education has an important role in maintaining personal hygiene, prevention of sexually transmitted disease, and understanding the effect of alcohol and smoking on sperm count. Our study has not found any difference between cases and controls in education as the two groups have the same percentage of educated participants.

Many studies have used the measurement of urinary DAP as a tool for determining OP pesticides exposure (13-18). As Yucra et al. (16) showed that occupation exposure of OP pesticides cannot be decided solely by OP metabolites measurement in urine, we have included both the determination of DAP in urine and measurement of pseudocholinesterase levels for labeling patient as OP-exposed. Hence, we may conclude that men with idiopathic abnormal semen analysis had high baseline exposure to OP pesticides. Li and Kannan (35) established the baseline levels of exposure to OP and pyrethroid pesticides among the population of several Asian countries. They concluded that India has the second-highest sum concentration of 11 pesticides in urine, next to Vietnam. Also, they found that daily intake of chlorpyrifos and parathion was high among the Indian population as compared to the population from other Asian countries. We got higher urinary levels of DEDTP and DETP in cases as compared to controls and these levels were significantly negatively associated with sperm concentrations, motility, and normal morphology. Perry et al. (10) concluded that men with lower semen quality had higher urinary DMP levels as compared to men with normal semen quality. Muñoz-Quezada et al. (36) concluded that urinary DAP levels were high in Chilean school children due to the presence of chlorpyrifos and phosmet residues in fruits.

There is a rising trend of male infertility among the population and for most of them, no detectable cause has been found. Hence, it has become the burning question and need of the hour to address what are the possible reasons for the decline in semen parameters? Because there is extensive use of OP pesticides in agriculture, its contamination in the food chain and its effect on sperm parameters, can sustain and a low dose of OP pesticides exposure be one of the causes for the decline of semen parameters among the Southern Indian population? In our study, we found that men with abnormal semen analysis had significantly higher OP pesticides exposure as compared to men with a normal semen analysis. OP-exposed men were farmers and from the rural population where they might be daily exposed to OP pesticides through food, water, and air, affecting their sperm parameters.

There were certain limitations in this study: i. We estimated acetylcholinesterase activity in serum instead of RBC. ii. There were six DAPs: DMP, DMTP, DMDTP, DEP, DETP, and DEDTP. Out of 6 metabolites, we estimated only 3 DAPs i.e. DMP, DETP, DEDTP due to lack of availability of remaining standards. iii. History of time of recent exposure was not known in our study. Hence, the impact of exposure on the spermatogenesis cycle was not estimated and there was not much information on the chemical insult window period in humans. iv. The seasonal variation of OP pesticides exposure was not considered in our study. v. Urinary DAP can be derived from pre-formed metabolites in the environment. vi. We have estimated semen analysis on one occasion. We were unable to repeat semen analysis hence characterization was not confirmed. vii. We didn’t estimate the hormonal changes in our study population. viii. This observational study has various unmeasured confounders like an instrumental variable, design, and so on. Due to time constraints, we have not addressed these confounders.

Though this is a pilot study, it explained a strong association between unintentional OP exposure and semen parameters. Hence, OP exposure status can be included as one of the investigations during the workup of men with an abnormal semen analysis. However, a future study including a larger sample size, more DAP metabolites, collection of more detailed information on demographic and socioeconomic parameters will be required to support our claim. 

## Conclusion

As OP pesticides exposure can occur through inhalation, ingestion, and so on, their subtle and chronic exposure is affecting various organs of the human body. The current study showed the effect of OP pesticides on semen parameters and concluded that men with idiopathic abnormal semen analysis had significantly higher OP pesticides exposure as compared to men with normal semen analysis. OP-exposed cases had higher urinary OP metabolites levels and more inhibition of pseudocholinesterase and acetylcholinesterase as compared to non-exposed cases pointing towards a severe degree of OP pesticides exposure. A higher percentage of OP-exposed men were farmers and from the rural area. 
